# Demographic and Physical Determinants of Unhealthy Food Consumption in Polish Long-Term Care Facilities

**DOI:** 10.3390/nu17061008

**Published:** 2025-03-13

**Authors:** Aia Ase, Jacek Borowicz, Kamil Rakocy, Edyta Krzych-Fałta, Bolesław Samoliński

**Affiliations:** 1 Department of the Prevention of Environmental Hazard, Allergology and Immunology, Faculty of Health Sciences, Medical University of Warsaw, 1a Banacha Street, 02-091 Warsaw, Poland; jacek.borowicz@wum.edu.pl (J.B.); boleslaw.samolinski@wum.edu.pl (B.S.); 2 Interdisciplinary Centre for Mathematical and Computational Modelling, University of Warsaw, 15/17 Tyniecka Street, 02-630 Warsaw, Poland; 3 Department of Basic Nursing, Medical University of Warsaw, 02-097 Warsaw, Poland; edyta.krzych-falta@wum.edu.pl

**Keywords:** unhealthy food consumption patterns, long-term care facility, demographic determinants, AI-based food classification, large language models, artificial intelligence in nutrition

## Abstract

**Background:** Unhealthy food consumption in long-term care facilities (LTCFs) contributes to poor health outcomes among residents. This study aimed to assess its prevalence, identify demographic and physical risk factors, and propose targeted interventions. **Methods:** A mixed-methods study (2017–2021) analyzed data from 1000 Polish LTCF residents (aged 35–105 years). Anthropometric measurements, bioimpedance analyses, dietary assessments, and physical activity records were collected. Food items were classified as “healthy” or “unhealthy” using an AI-based Large Language Model (LLM), applying WHO guidelines and the NOVA classification system. Logistic regression and chi-square tests assessed associations between unhealthy food consumption and marital status, education level, mobility aid use, and portion control. **Results:** Unhealthy food consumption prevalence was 15.6%. Married residents had significantly higher rates than unmarried individuals (22.6% vs. 14.3%, *p* < 0.01). Lower educational attainment correlated with increased risk (partial primary education: 34.7% vs. tertiary education: 8.1%). Mobility aid users exhibited elevated consumption (cane: 34.6%; walker: 22.6%). Poor portion control showed the strongest association (OR = 3.2, 95% CI: 1.8–5.7). **Conclusions:** Marital status, educational disparities, mobility limitations, and portion control were key modifiable risk factors. Findings suggest the need for targeted nutrition programs, caregiver education, and policy reforms to improve dietary literacy and meal portioning. Future research should validate AI-based food classification methods, assess long-term intervention outcomes, and expand studies to diverse LTCF settings. These findings align with Poland’s National Health Programme and provide actionable insights for global LTCF populations.

## 1. Introduction

The global increase in aging populations has led to a growing demand for long-term care services [1], necessitating a deeper understanding of the complex interactions between demographic factors—such as age, gender, and socioeconomic status—and the care environment in shaping dietary behaviors [2,3]. These interactions have profound implications for addressing malnutrition, managing chronic diseases, and enhancing the quality of life among institutionalized older adults, who frequently experience dietary restrictions and health challenges [4,5].

### 1.1. Nutritional Challenges in Long-Term Care Facilities

Dietary habits in long-term care facilities (LTCFs) are a critical determinant of residents’ health, particularly given the high prevalence of age-related metabolic changes and chronic diseases in this population [6]. Although institutional meal programs typically adhere to standardized nutritional guidelines [7], the storage and consumption of externally sourced foods—commonly referred to as “cabinet foods”—has emerged as an understudied risk factor for obesity, diabetes, and cardiovascular disease [8,9]. Recent multinational studies indicate that 15–30% of LTCF residents regularly consume ultra-processed foods (UPFs) as classified by the NOVA system, with consumption patterns varying significantly based on socioeconomic and cultural factors [10,11].

### 1.2. Demographic and Physical Determinants of Dietary Patterns

LTCF populations exhibit distinct demographic characteristics, with a predominance of female residents and a mean age exceeding 80 years [12]. Socioeconomic disparities further shape dietary behaviors, particularly in care settings where access to nutritious food is constrained by affordability and institutional policies [13]. Marital status plays a key role in dietary choices, as family visits often introduce calorie-dense “comfort foods” into residents’ diets [9]. Additionally, educational attainment influences nutritional literacy, with individuals having ≤8 years of formal education showing a 3.4-fold increased likelihood of unhealthy snacking compared to their college-educated peers [14,15]. Physical limitations further exacerbate dietary risks, with mobility aid users experiencing a 27% reduction in access to fresh foods due to reliance on caregivers for food procurement, as documented in German and Canadian LTCFs [16].

### 1.3. Defining and Assessing Unhealthy Dietary Consumption

The classification of “unhealthy” food consumption remains methodologically challenging. While WHO guidelines define unhealthy foods based on thresholds for free sugars (>10% total energy), saturated fats (>10%), and sodium (>2 g/day) [17], the NOVA classification provides a complementary framework by identifying UPFs based on industrial processing characteristics [18]. Recent AI-driven dietary classification systems have demonstrated high concordance with expert dietitian assessments, presenting a novel opportunity to enhance dietary evaluation while mitigating biases associated with self-reported food intake [19].

### 1.4. Gaps in Current Interventions and Study Objectives

Despite growing recognition of these challenges, interventions targeting dietary behaviors in LTCFs have shown mixed effectiveness. While staff training programs in Canadian LTCFs have improved dietary quality and reduced UPF consumption [20,21], family-centered nutritional interventions in Italian settings have achieved only modest long-term compliance [22]. While previous studies have explored individual factors affecting dietary choices in LTCFs [13,23,24,25], comprehensive analyses integrating demographic, socioeconomic, and physical determinants remain scarce. Notably, no prior research has leveraged AI-powered classification techniques to provide an objective, large-scale dietary assessment, filling a critical methodological gap in geriatric nutrition research.

This national cohort study represents a methodological advancement in geriatric nutrition research by introducing three key innovations:

AI-powered nutritional phenotyping using a Large Language Model (LLM) for objective classification of cabinet foods, overcoming recall biases prevalent in traditional dietary assessments [26,27,28].

Interaction effect analysis between marital status, mobility limitations, and educational disparities, factors that are underexplored in European LTCF populations.

The study’s policy-ready risk stratification provides a model for targeting high-risk groups in dietary interventions, aligning with national health initiatives such as Poland’s National Health Programme 2025–2030 and offering a scalable framework for other aging populations worldwide.

Given the significant health implications of dietary patterns in LTCFs—where inadequate nutrition is strongly linked to chronic disease burden and reduced quality of life—developing evidence-based interventions that account for both demographic and physical determinants is critical [29,30,31,32]. By integrating epidemiological rigor with computational nutrition science, this study provides a framework for improving dietary management in institutional care settings worldwide.

## 2. Materials and Methods

This study was founded by the Ministry of Health (Poland) under the National Health Program 2016–2020 [33]. Ethical approval was obtained from the Institutional Ethical Review Board at the Medical University of Warsaw (Approval No. AKBE/212/2017, 12 December 2017).

**Figure 1:**
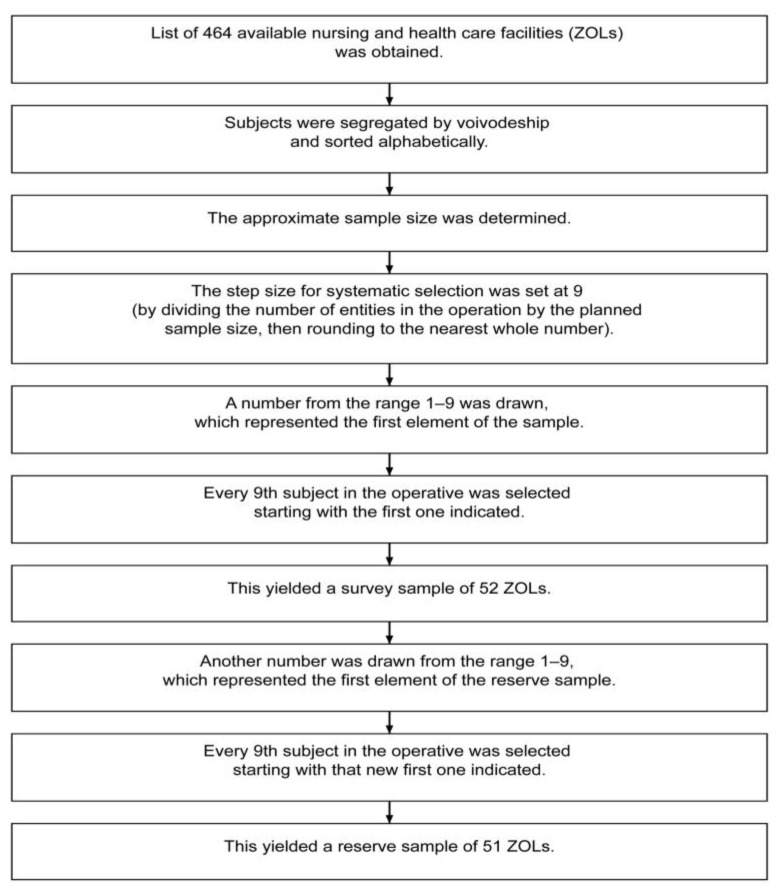
The method of selecting nursing and health care facilities.

The mixed-methods study was conducted in two phases: a qualitative phase (2017 and January 2019) and a quantitative phase (2018–2021). Data were prospectively collected from 1000 residents in long-term care facilities (LTCFs) across Poland. Facilities were randomly selected using stratification based on the National Health Fund’s Integrated Patient Information System (see Figure 1).

**Figure 2:**
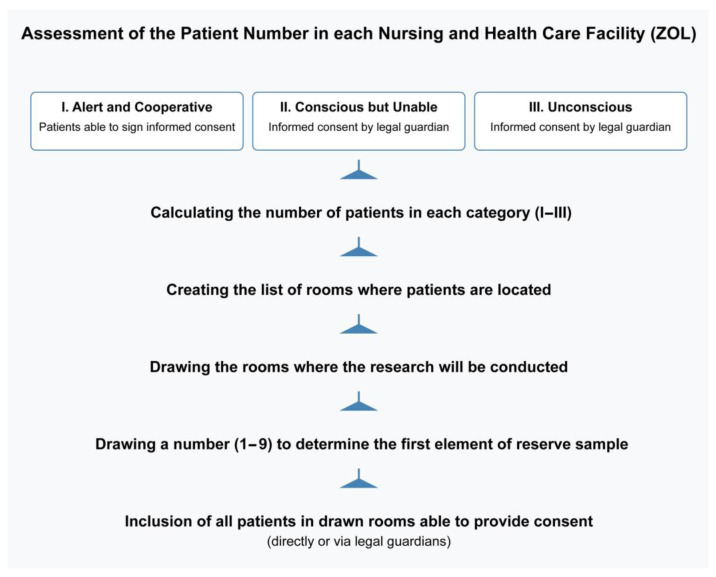
The method of the enrollment of the patients.

The study population included adults residing in LTCFs (ZOL) who required long-term care due to chronic conditions, regardless of age. The method of selecting patients is shown in Figure 2.

The first step distinguishes patients based on whether they can sign for themselves or require a guardian’s consent. This is a practical approach in healthcare settings, as it ensures all ethical and legal requirements for informed consent are met (especially for unconscious or cognitively impaired patients).

The final step makes it clear that all patients in the selected rooms who can provide consent (or have legal guardian consent) are included. This maximizes the inclusion of eligible participants once a room is chosen, without adding a second round of individual randomization.

Patients who remained unconscious and those who did not sign informed consent were not eligible for the study. Individuals with a fluctuating state of consciousness were included in the study—however, only data acquired when conscious were analyzed.

Each participant was observed for 3 consecutive days. The research material included anthropometric data, bioimpedance analyses, physical activity, prevalent diseases, and the quality and quantity of meals consumed. Our study systematically examined the collection, analysis, and categorization of food products stored in the cabinets of residents at LTCFs. The methodology followed a structured approach.

To gather relevant information, open-ended questions were utilized. These questions aimed to identify the types of food products stored in the cabinets of LTCFs’ residents. This qualitative approach enabled a thorough understanding of dietary habits and the variety of food items consumed by this population.

The collected data were analyzed using Claude 3.7 Sonnet, an advanced AI model released on 24 February 2025, and utilized in this study on 2 March 2025.

The Claude model was selected due to its widespread adoption in European research institutions for qualitative data analysis, making it well-suited for the study’s methodological requirements. For instance, the European Parliament utilizes Claude to enhance accessibility to its archives, streamlining document search and analysis processes [34]. With its extended context window and ability to process long-form, unstructured data, Claude demonstrates strong capabilities in text interpretation, making it particularly effective for analyzing qualitative dietary information [35].

Food items were classified as “healthy” or “unhealthy” using this advanced Large Language Model (LLM), guided by established nutritional criteria from both WHO guidelines [36,37] and the NOVA classification system [38], with a total of 1543 food products analyzed through this process.

The classification process was conducted in two sequential steps, integrating both the NOVA classification system and WHO nutritional guidelines to ensure comprehensive dietary assessment:

Step 1: Initial Categorization Based on Processing Criteria (NOVA System)

The classification began by identifying ultra-processed foods (UPFs) using the NOVA classification system, which categorizes foods based on the extent and purpose of industrial processing. UPFs, typically energy-dense and nutrient-poor, were classified as unhealthy due to their formulation and impact on overall diet quality.

Step 2: Nutritional Evaluation Based on WHO Guidelines

After the initial processing-based classification, all items categorized as healthy in Step 1 were further evaluated using WHO nutritional thresholds. Foods exceeding the WHO-defined limits for free sugars (>10% of total energy), saturated fats (>10%), or sodium (>2 g/day) were reclassified as unhealthy.

A standardized two-step prompting framework was developed to ensure an accurate and systematic dietary classification. The AI model was instructed to act as a dietary assistant, analyzing food items based on both processing characteristics.

The model output included product name, weight (g). total caloric content, final classification (healthy/unhealthy).

The finalized prompt was as follows:

“Prepare dictionary with healthy and unhealthy food for each file and check it with this prompt: Step 1 Act as a helpful dietary assistant. Classify the following food products as ‘healthy’ or ‘unhealthy’ based on established nutritional criteria. For this classification, consider foods unhealthy if they are identified as ultra-processed according to the NOVA classification system. For each product, provide details in the following format: {product, weight (g), calories, classification}. Finally, sum the total calories for each category. Products: [list of products] Step 2—for all in HEALTHY: Act as a helpful dietary assistant. Classify the following food products as ‘healthy’ or ‘unhealthy’ based on established nutritional criteria. For this classification, consider foods unhealthy if they exceed WHO thresholds for free sugars (>10% of total energy), saturated fats (>10%), or sodium (>2 g/day). For each product, provide details in the following format: {product, weight (g), calories, classification}. Finally, sum the total calories for each category. Products: [list of products]”

Although LTCFs typically house an older population, our inclusion criteria allowed for younger residents due to the presence of chronic conditions warranting institutional care. To address potential age-related heterogeneity, sensitivity analyses were performed, and age was included as a covariate in regression models to control for its effect on dietary behaviors.

Data were analyzed using standard statistical methods:

- Categorical Variables: Associations between dietary behaviors and demographic/physical factors were assessed using chi-square tests. When cell counts were below the threshold (i.e., fewer than 5 observations per cell), Fisher’s exact test was employed to ensure robustness of the statistical inference.
- Regression Analysis: Binary logistic regression was selected due to the dichotomous nature of the dependent variable (healthy/unhealthy). Other regression models, such as multinomial or ordinal regression, were considered but deemed unnecessary since the classification system did not involve three or more ordered categories. Predictor variables included marital status, education level, and mobility aid use, with age incorporated as a covariate to control for potential confounding. Model performance was evaluated using Cox and Snell and Nagelkerke R^2^ values to assess explanatory power. Residual diagnostics confirmed the appropriateness of logistic regression for this dataset.

## 3. Results

The age range of the individuals was 35–105 in females and 41–97 in males. Women generally have a longer life expectancy than men, leading to a higher proportion of elderly females in LTCFs. This demographic shift is particularly pronounced in populations over the age of 80 [39]. However, most of the study population consisted of older subjects, with a median of 81. The number of participants within each gender category amounted to 692 in females and 308 in males. The gender distribution in the study reflects demographic trends commonly observed in LTCFs [2].

Several demographic factors were associated with unhealthy food consumption.

### 3.1. Marital Status and Unhealthy Food Consumption

Married residents were more likely to consume unhealthy foods compared to their unmarried counterparts (22.6% vs. 14.3%, see Table 1).

Of the total sample (N = 693), 587 participants were unmarried (84.7%), and 106 were married (15.3%). Among those who consumed unhealthy food (n = 108), 77.8% were unmarried and 22.2% were married. The data suggest that married individuals were more likely to consume unhealthy food than their unmarried counterparts. This trend may reflect cultural or familial practices where family members bring comfort foods during visits. Family members often provide snacks or treats as gifts for residents, prioritizing emotional comfort over nutritional value. However, these high-calorie items may inadvertently harm patients’ health.

The relationship between marital status and unhealthy food consumption was examined using a chi-square test of independence. A significant association was found between these variables (χ^2^(1, N = 693) = 4.737, *p* = 0.030). Among unmarried individuals, 14.3% reported consuming unhealthy food, compared to 22.6% among married individuals. Fisher’s test confirmed this relationship (*p* = 0.041, two-sided). The minimum expected cell count was 16.52, meeting the assumption for chi-square analysis as no cells had expected counts less than 5.

### 3.2. Education and Unhealthy Food Consumption

The prevalence of unhealthy food consumption varied notably across educational levels (see Table 2). The highest proportion was observed among those with partial primary education (34.7%), while the lowest rates were found among individuals with basic vocational education (10.3%) and those with tertiary education (11.7%). Among participants with primary/high school education, 13.3% reported consuming unhealthy food, while the rate was 17.8% for those with secondary education. In cases where the tutor’s knowledge was lacking (n = 37), 10.8% of individuals reported unhealthy food consumption. This suggests that nutritional awareness plays a significant role in shaping dietary choices.

A chi-square test of independence revealed a significant association between educational level and unhealthy food consumption (χ^2^ (5, N = 663) = 19.166, *p* = 0.002). The minimum expected cell count was 5.69, satisfying the assumptions for chi-square analysis as no cells had expected counts less than 5.

Of the total sample (N = 663), the largest educational groups were primary/high school (n = 203, 30.6%) and secondary education (n = 197, 29.7%), while tertiary education (n = 60, 9.0%) and lack of tutor knowledge (n = 37, 5.6%) represented smaller proportions. Overall, 15.4% of the sample reported consuming unhealthy food, regardless of educational level.

### 3.3. Mobility Aid Use and Unhealthy Food Consumption

The study also found a link between physical mobility aids and unhealthy food consumption. Residents using canes (34.6%) or walkers (22.6%) were more likely to consume unhealthy foods than non-users (see Table 3 and Table 4). Mobility aid users may face barriers to accessing healthier food options or rely on others for food provision, which could explain their higher consumption rates of unhealthy products.

The association between mobility aid (cane) use and unhealthy food consumption was examined using Fisher’s exact test, given that 25% of cells had expected counts less than 5 (minimum expected count = 4.06). The analysis revealed a significant relationship (*p* = 0.012, two-sided). Among individuals who did not use a cane (n = 665), 14.9% reported consuming unhealthy food, compared to 34.6% among cane users (n = 26).

The chi-square test results (χ^2^(1, N = 691) = 7.385, *p* = 0.007) align with Fisher’s exact test, suggesting that cane users were more likely to consume unhealthy food than non-users. However, these findings should be interpreted cautiously, given the relatively small number of cane users in the sample (3.8% of total participants).

The association between walker use and unhealthy food consumption was analyzed using a chi-square test of independence. A marginally significant relationship was found (χ^2^(1, N = 691) = 3.938, *p* = 0.047), though this relationship became non-significant when applying the continuity correction (*p* = 0.067). Fisher’s exact test yielded similar results (*p* = 0.064, two-sided). Among walker users (n = 93), 22.6% reported consuming unhealthy food, compared to 14.5% among non-users (n = 598).

The analysis met the assumptions for chi-square testing, with a minimum expected cell count of 14.54 and no cells having expected counts less than 5. Walker users represented 13.5% of the total sample. While the data suggest a trend toward higher unhealthy food consumption among walker users, the evidence for this association is relatively weak, given the borderline significance levels across different statistical tests.

### 3.4. Impact of Portion Control on Unhealthy Food Consumption

Poor portion control was another contributing factor, emphasizing the need for education on portion sizes and balanced diets (see Table 5).

A chi-square test of independence revealed a significant association between portion size assessment and unhealthy food consumption (χ^2^(2, N = 313) = 9.440, *p* = 0.009). However, one cell (16.7%) has an expected count of less than 5 (minimum expected count = 1.94), so these results should be interpreted cautiously.

Among participants with good portion size (n = 242), 20.2% reported consuming unhealthy food, compared to 38.1% of those with average portion size (n = 63) and 37.5% of those with bad portion size (n = 8). Most participants were assessed as having a good portion size (77.3% of the sample), while only 2.6% were classified as having a bad portion size.

The linear-by-linear association test (χ^2^(1) = 8.453, *p* = 0.004) suggests a trend toward increased unhealthy food consumption as portion size assessment worsens. However, given the small number of participants in the “bad” portion size category, further research with a larger sample size would be needed to confirm this relationship.

### 3.5. Predictors of Unhealthy Food Consumption: The Role of Marital Status, Education, and Mobility Aid Use

A binary logistic regression was performed to assess the effects of marital status, education level, and mobility aids (walker and cane use) on the likelihood of unhealthy food consumption. The model explained the variance in unhealthy food consumption between 4.8% (Cox and Snell R^2^) and 8.4% (Nagelkerke R^2^), see Table 6 and Table 7.

Marriage was significantly associated with unhealthy food consumption (OR = 0.484, *p* = 0.009), with unmarried individuals being less likely to consume unhealthy food. Education level was also a significant predictor (*p* = 0.004), with participants having partial primary education showing higher odds of unhealthy food consumption than those lacking tutor knowledge (OR = 4.108, *p* = 0.022). The remaining educational categories (primary/high school, basic vocational, secondary education, and tertiary education) did not show significant associations with unhealthy food consumption (all *p* > 0.05).

Mobility aid use was significantly associated with unhealthy food consumption. Non-users of walkers were less likely to consume unhealthy food than walker users (OR = 0.260, *p* = 0.003). Similarly, non-users of canes showed lower odds of unhealthy food consumption than cane users (OR = 0.563, *p* = 0.047).

## 4. Discussion

This study provides valuable insights into the demographic and physical factors influencing unhealthy food consumption among residents of Polish LTCFs, identifying significant associations with marital status, education level, and mobility aid use.

While these findings contribute to the broader understanding of geriatric nutrition and long-term care, certain limitations must be acknowledged. The duration of dietary monitoring in our study was relatively short (covering 3 days); as a result, the data may not capture usual long-term dietary behaviors. The study’s focus on Polish LTCFs may limit the generalizability of the results to other cultural or institutional contexts [40,41]. To ensure broader applicability, future research should examine whether similar patterns emerge in diverse settings.

It is essential to consider several limitations and to interpret the results within the wider context of dietary behaviors in institutional care.

### 4.1. Marital Status and Unhealthy Food Consumption

Married residents were significantly more likely to consume unhealthy foods than unmarried individuals (22.6% vs. 14.3%, *p* = 0.030). This association could be attributed to family members visiting LTCF residents, often prioritizing emotional comfort over nutritional value by bringing high-calorie snacks and processed foods as gifts. This aligns with previous studies indicating that social connections influence food choices, sometimes reinforcing unhealthy dietary patterns.

However, it is also crucial to consider alternative explanations. For example, married residents may have different pre-existing dietary habits that they maintain even within the LTCF, or their family members may be more attentive to their perceived desires, regardless of nutritional value. Educational campaigns targeting family members and caregivers could help promote healthier alternatives without compromising the emotional benefits of shared meals. These campaigns could emphasize the long-term health consequences of seemingly harmless treats, and offer practical suggestions for nutritious and enjoyable alternatives [42].

Future research should also investigate the specific types of unhealthy foods most often consumed by married residents and explore the cultural and social meanings associated with these foods.

### 4.2. Educational Level and Nutritional Awareness

Education level was also a strong predictor of unhealthy food consumption. Residents with lower educational attainment (e.g., partial primary education) were more likely to consume unhealthy foods (34.7% vs. 8.1% among tertiary-educated residents, *p* = 0.002). This suggests that nutritional literacy plays a significant role in dietary choices.

However, the relationship between education and dietary choices is likely multifaceted. It is also possible that individuals with lower educational attainment have experienced a lifetime of limited access to healthy food options and may not have the financial resources to afford nutritious choices, regardless of their knowledge [43,44].

Future strategies should include simplified, visually guided nutrition programs tailored to residents with lower education levels to enhance their understanding of healthy eating habits. These programs should consider the specific cultural and socioeconomic contexts of the residents.

### 4.3. Mobility Aid Use and Dietary Choices

A significant association was found between cane use and unhealthy food consumption (*p* = 0.012), suggesting that mobility limitations may impact dietary access and choices. However, given the small number of cane users in our sample (3.8% of total participants, n = 26), this finding should be interpreted with caution. Although walker users also exhibited a trend toward increased unhealthy food consumption (22.6% vs. 14.5% among non-users), this association was only borderline significant (*p* = 0.047 before correction, *p* = 0.067 after), highlighting the need for further research with larger samples.

One possible explanation for these trends is that residents relying on mobility aids may face barriers to accessing healthier food options and rely on caregivers or family members for food provision. Furthermore, qualitative research could explore the experiences of residents with mobility limitations, gaining a deeper understanding of the challenges they face in accessing healthy food.

Prior research has indicated that limited mobility often restricts dietary autonomy, making residents more likely to consume pre-packaged or calorie-dense snacks rather than fresh, nutrient-dense foods [45]. Future interventions should explore targeted meal assistance programs to support residents with mobility impairments, ensuring they have access to a variety of nutritious and appealing food choices. LTCFs should implement strategies to enable mobility-impaired residents to make informed food choices.

### 4.4. Portion Size Control and Dietary Habits

Residents with poor portion control were significantly more likely to consume unhealthy foods (χ^2^(2, N = 313) = 9.440, *p* = 0.009). However, caution is needed in interpreting these results due to the small sample size in the “bad” portion size category (n = 8). The observed trend suggests that portion misjudgment may contribute to excessive intake of unhealthy foods, reinforcing the need for portion control education within LTCFs. In fact, prior studies report that caregivers misjudge portion sizes in more than half of observations (errors ~56% for immediate estimates, worsening with delayed recording) [46].

Future interventions should incorporate behavioral strategies to promote mindful eating and address underlying psychological factors that contribute to poor portion control.

### 4.5. Methodological Considerations: The Role of AI in Dietary Assessment

A key innovation of this study was the use of an LLM to classify food items as “healthy” or “unhealthy,” offering an objective and scalable method for dietary assessment that potentially mitigates biases associated with self-reported data. Our findings support the notion that embracing AI tools in dietetics is not only feasible but potentially transformative. Our results align with the growing body of research advocating AI-assisted dietary assessments in real-world settings [47].

However, several limitations must be considered. The reliance on self-reported data or cabinet inventories may not fully capture all dietary habits, potentially leading to underreporting or overreporting certain behaviours [48]. The accuracy of the LLM depends on the completeness and quality of the food product information available to the model [49], and the classification relies on predefined nutritional criteria (WHO guidelines and the NOVA system), which may not capture the full nuances of individual dietary needs [50,51].

Notably, our study employed Claude 3.7—one of many available LLMs—to perform the food classification. As such, it is uncertain whether other LLMs would achieve comparable accuracy in segregating food items. Moreover, the rapid pace of advancements and frequent upgrades in LLM technology suggest that the performance of Claude 3.7 today might differ from that of future versions or entirely new models, posing challenges for reproducibility in a scientific context [52,53,54].

Direct comparisons between studies are difficult due to differences in datasets and evaluation metrics [46], but these findings collectively strengthen the evidence that AI can deliver accurate dietary assessments in geriatric populations.

Future research should validate the LLM-based classification system against traditional dietary assessment methods (e.g., dietary recalls or food diaries) and refine the nutritional criteria to account for individual variations. Additionally, exploring the integration of LLMs with culturally specific food databases and recipes could enhance the accuracy and generalizability of dietary assessments across diverse populations.

It would also be valuable to explore integration with other smart systems—for example, combining intake data with electronic medical records could enable predictive analytics to identify residents at risk of decline. Successful integration of AI in nutrition programs will require training end-users (health professionals and the public) to use these tools effectively. Simultaneously, investment in technological infrastructure is needed to support widespread use—for example, expanding internet access in underserved areas and providing community health centers with the necessary devices or technical support. These investments align with the WHO’s Global Strategy on Digital Health, which calls for leveraging digital innovations to improve health outcomes while ensuring no one is left behind [55].

## 5. Conclusions

This study highlights the significant influence of demographic and physical factors on unhealthy food consumption among residents of Polish LTCFs. The findings underscore the need for targeted interventions, such as nutrition education programs, family engagement initiatives, and tailored meal assistance strategies, to enhance dietary health and overall well-being in long-term care settings.

Additionally, this study affirms the scientific validity and practical potential of AI-based dietary assessment in geriatric nutrition. While AI-driven approaches offer promising advancements in dietary monitoring, their limitations must be carefully addressed to ensure accuracy and applicability across diverse populations.

Future research should focus on validating AI-based food classification systems, exploring the underlying mechanisms of dietary behaviors, and evaluating the long-term effectiveness of targeted interventions. Addressing these research gaps and refining existing methodologies will enable the development of more effective, evidence-based strategies to promote healthy aging and improve the quality of life for LTCF residents worldwide.

## Figures and Tables

**Table 1:** Marital status and prevalence of unhealthy food consumption among LTCF residents.

			Married		Total
			No	Yes	
Unhealthy food		Count	503	82	585
	No	% within Married	85.7%	77.4%	84.4%
		Count	84	24	108
	Yes	% within Married	14.3%	22.6%	15.6%
	Count		587	106	693
Total	% within Married		100.0%	100.0%	100.0%

**Table 2:** Educational attainment and unhealthy food consumption among LTCF residents.

			Unhealthy Food		Total
			No	Yes	
Education	Partial primary	Count	32	17	49
		% within Education	65.3%	34.7%	100.0%
	Primary/high school	Count	176	27	203
		% within Education	86.7%	13.3%	100.0%
	Basic vocational	Count	105	12	117
		% within Education	89.7%	10.3%	100.0%
	Secondary education (general or technical)	Count	162	35	197
		% within Education	82.2%	17.8%	100.0%
	Tertiary (bachelor’s, engineering, master’s degree)	Count	53	7	60
		% within Education	88.3%	11.7%	100.0%
	Lack of knowledge of the tutor	Count	33	4	37
		% within Education	89.2%	10.8%	100.0%
Total		Count	561	102	663
		% within Education	84.6%	15.4%	100.0%

**Table 3:** Mobility aid use (cane) and unhealthy food consumption among LTCF residents.

			Unhealthy Food		Total
			No	Yes	
5. The patient moves: (2) with the help of a cane	NO	Count	566	99	665
		% within 5. The patient moves: (2) with the help of a cane	85.1%	14.9%	100.0%
	YES	Count	17	9	26
		% within 5. The patient moves: (2) with the help of a cane	65.4%	34.6%	100.0%
Total		Count	583	108	691
		% within 5. The patient moves: (2) with the help of a cane	84.4%	15.6%	100.0%

**Table 4:** Mobility aid use (walker) and unhealthy food consumption among LTCF residents.

			Unhealthy Food		Total
			No	Yes	
5. The patient moves: (4) with a walker	NO	Count	511	87	598
		% within 5. The patient moves: (4) with a walker	85.5%	14.5%	100.0%
	YES	Count	72	21	93
		% within 5. The patient moves: (4) with a walker	77.4%	22.6%	100.0%
Total		Count	583	108	691
		% within 5. The patient moves: (4) with a walker	84.4%	15.6%	100.0%

**Table 5:** Impact of portion control on unhealthy food consumption in LTCF.

			Unhealthy Food		Total
			No	Yes	
64. portion size	1—good	Count	193	49	242
		% within 64. portion size	79.8%	20.2%	100.0%
	2—average	Count	39	24	63
		% within 64. portion size	61.9%	38.1%	100.0%
	3—bad	Count	5	3	8
		% within 64. portion size	62.5%	37.5%	100.0%
Total		Count	237	76	313
		% within 64. portion size	75.7%	24.3%	100.0%

**Table 6:** Model summary for stepwise logistic regression analysis.

Step	−2 Log Likelihood	Cox and Snell R Square	Nagelkerke R Square
1	536,567 ^a^	0.048	0.084

^a^ Estimation terminated at iteration number 5 because parameter estimates changed by less than 0.001.

**Table 7:** Model fit statistics for binary logistic regression predicting unhealthy food consumption.

		B	S.E.	Wald	df	Sig.	Exp(B)
Step 1 ^a^	Married(No)	−0.726	0.277	6.887	1	0.009	0.484
	Education			17.588	5	0.004	
	Partial primary	1.413	0.619	5.218	1	0.022	4.108
	Primary/high school	0.139	0.576	0.059	1	0.809	1.150
	Basic vocational	−0.210	0.618	0.115	1	0.734	0.811
	Secondary education (general or technical)	0.459	0.568	0.653	1	0.419	1.582
	Tertiary (bachelor’s, engineering, master’s degree)	−0.004	0.672	0.000	1	0.995	0.996
	The patient moves: (4) with a walker—No	−1.348	0.457	8.680	1	0.003	0.260
	The patient moves: (2) with the help of a cane—No	−0.574	0.289	3.941	1	0.047	0.563
	Constant	0.344	0.805	0.183	1	0.669	1.411

## Data Availability

The data that support the findings of this study are available from the corresponding author upon reasonable request due to legal reasons.
